# Workmen's Contribution to Leeds Infirmary

**Published:** 1888-03-24

**Authors:** Fred. R. Spark

**Affiliations:** Leeds


### THE EDITOR'S LETTER-BOX.

[Our Correspondents are reminded that prolixity is a great bar to publication, and that brevity of style and conciseness of statement greatly facilitate early insertion.]

### WORKMEN'S CONTRIBUTIONS TO LEEDS INFIRMARY.

As your report on the figures given at the annual meeting of the Leeds Infirmary may convey a wrong idea as to the workpeople's contributions during 1887, kindly allow me to show how I make up the total to about £4,847:

| | £ | s. | d. |
|---|---|---|---|
| Workpeople's contributions in Leeds | £3,694 | 14 | 11 |
| Workpeople's contributions for two ambulances | 328 | 0 | 0 |
| Workpeople's contributions in Leeds out-districts | 574 | 1 | 4 |
| From trade and friendly societies | 250 | 0 | 0 |
| | £4,846 | 16 | 3 |

From the same source in 1886 the contributions were £2,613 7*s*. 6*d*. FRED. R. SPARK.

Leeds.

